# Medical intelligence using PPG signals and hybrid learning at the edge to detect fatigue in physical activities

**DOI:** 10.1038/s41598-024-66839-8

**Published:** 2024-07-12

**Authors:** Ping Liu, Yazhou Song, Xuan Yang, Dejuan Li, M. Khosravi

**Affiliations:** 1 Department of Physical Education and Teaching, Hebei Finance University, Baoding, 071051 China; 2 Faculty of Sport Sciences & Recreation, Universiti Teknologi MARA (UiTM), 40450 Shah Alam, Selangor Malaysia; 3 Graduate School, Angeles University Foundation, 2009 Angeles, Philippines; 4 School of Medicine, Shiraz University of Medical Sciences, Shiraz, Iran; 5 Shandong Provincial University Laboratory for Protected Horticulture, Weifang University of Science and Technology, Shouguang, Weifang, 262700 Shandong China

**Keywords:** Fatigue detection, Deep learning, Photoplethysmography signals, Physiological signal, ResNetCNN, Xception architecture, Bidirectional long short-term memory, Health services, Biomedical engineering

## Abstract

The educational environment plays a vital role in the development of students who participate in athletic pursuits both in terms of their physical health and their ability to detect fatigue. As a result of recent advancements in deep learning and biosensors benefitting from edge computing resources, we are now able to monitor the physiological fatigue of students participating in sports in real time. These devices can then be used to analyze the data using contemporary technology. In this paper, we present an innovative deep learning framework for forecasting fatigue in athletic students following physical exercise. It addresses the issue of lack of precision computational models and extensive data analysis in current approaches to monitoring students’ physical activity. In our study, we classified fatigue and non-fatigue based on photoplethysmography (PPG) signals. Several deep learning models are compared in the study. Using limited training data, determining the optimal parameters for PPG presents a significant challenge. For datasets containing many data points, several models were trained using PPG signals: a deep residual network convolutional neural network (ResNetCNN) ResNetCNN, an Xception architecture, a bidirectional long short-term memory (BILSTM), and a combination of these models. Training and testing datasets were assigned using a fivefold cross validation approach. Based on the testing dataset, the model demonstrated a proper classification accuracy of 91.8%.

## Introduction

Humans experience fatigue as a subjective phenomenon. Fatigue affects the brain’s response time negatively, resulting in decreased performance, less attention and judgement, as well as unpleasant emotional responses. The physical demands of sport, such as long hours of training, and the mental stress of competition can lead to sport-related fatigue in students^1^. Furthermore, students may feel overwhelmed if they have to balance studies and sports^2^. In addition to dehydration, poor nutrition, and insufficient sleep, exercise-induced fatigue can be caused by a variety of factors. Furthermore, fatigue can be exacerbated by overusing certain muscles or joints as well as by not warming up or stretching properly before exercise^3^. People across diverse occupations are susceptible to the effects of fatigue. Insufficient sleep and an overwhelming workload for student fatigue due to sports are two prominent factors that contribute to exhaustion; however, chronic fatigue syndrome and cancer may also play a role^4^. Monitoring and being aware of one’s level of weariness consistently over time not only promotes more effective management of exhaustion, but also reduces the risk of other health issues caused by the presence of other medical conditions and accidents.

In contemporary times, heart rate variability (HRV) has gained prominence as a metric for assessing fatigue^5,6^. Numerous research studies use conductive glue as the conductor in order to capture the electrocardiogram (ECG) at three distinct anatomical sites in order to compute HRV indices. A solitary electrode patch is applied firmly to the breast region exposed to clothing to address the potential consequences of clothing. In addition to the thoracic region and the manual extremities, measurements can also be obtained there. Then, peak detection techniques are used to calculate heart rate variability (HRV) values by determining the durations between consecutive heartbeats^7^. Because of power noise and electromagnetic interference (EMI), the HRV research was compromised due to alterations in ECG results during measurement^8^. Electrocardiogram (ECG) signals are susceptible to contamination by electric and magnetic interference (EMI), which introduces unnecessary information. When examining heart rate variability, this phenomenon makes it difficult to accurately measure intervals and conceal R-wave peaks. Furthermore, environmental factors affected the evaluation of HRV. There are several potential reasons why the correlation between HRV indices and fatigue could be concealed, including changes in breathing patterns, parasympathetic nerve activity initiated by relaxation, and emotional fluctuations^9,10^. HRV measures can be affected by several factors, such as the level of physical exertion^11^, drug use^12^, psychological state^13^, and preexisting medical conditions^14^.

As optoelectronic and electronic technology has progressed, PPG has become a popular method for assessing the vascular health of blood vessels. The determination of different physiological parameters, including epileptic seizure classification, biometric identification, blood oxygen saturation levels (SpO_2_), blood pressure, and heart rate, can be accomplished by employing suitable preprocessing techniques on the PPG signal, in conjunction with the use of artificial intelligence algorithms^15–19^. It is therefore highly promising to use the PPG signal. There is no need for complex algorithms or expensive instruments to assess PPG. There are no inherent risks associated with photoplethysmography (PPG) and no diseases are spread in the process of detecting a PPG signal. Human physiological signals can be assessed using PPG signals in a simple and effective way, offering individuals a non-invasive method of examination. Researchers have used PPG in the past to determine the association between HRV and fatigue^20,21^. However, very few studies have used PPG to assess tiredness. While using PPG to calculate HRV, several issues were found^9,10^.

The stimulation of nerve terminals and subsequent weariness can be attributed to insufficient blood flow^22,23^, since this condition causes metabolic waste to build up inside the blood vessels. PPG can be applied in this context to analyze temporal changes in intravascular blood volume and quantify microcirculation and intravascular fluid volume^24,25^. Using PPG signals, this study evaluated and developed fatigue indices that take the dicrotic peak into account when assessing tiredness.

Edge intelligence and deep learning are able to detect fatigue in PPG signals quickly and accurately, as they are able to process large amounts of data quickly and analyze them using complex algorithms. Additionally, edge intelligence and deep learning are able to take into account multiple factors, such as heart rate variability, to detect fatigue more accurately than traditional methods of fatigue detection. This makes them ideal for applications where fatigue detection is required in real-time, such as in aviation, transportation, and healthcare. Additionally, edge intelligence and deep learning are cost-effective and energy-efficient, making them an ideal solution for many applications.

**Figure 1:**
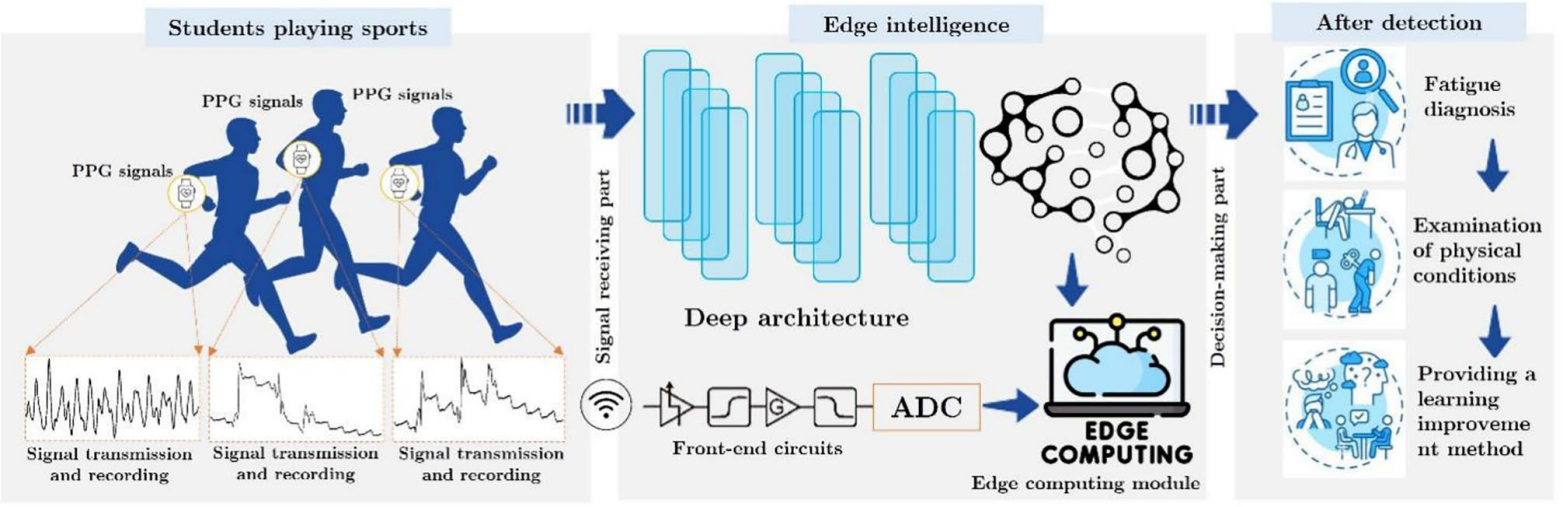
The framework shows a view of the overall performance of the proposed approach, displaying how techniques such as edge intelligence and deep learning can be employed to detect student fatigue during sports.

In this study, we examined a number of indicators of student fatigue due to sports, such as HRV, a newly constructed fatigue index, and objective fatigue measures. Hence, the aim was to identify the most suitable indices of student fatigue due to sports and conduct comprehensive research and extensive assessments of fatigue. With regard to enhancing classification accuracy in situations characterized by a scarcity of data, the present study utilized a restricted dataset to devise a deep learning methodology that evaluates network design and parameter allocation. It is imperative to explore methodologies for determining optimal parameter values for the models to resolve this issue. Additionally, it is expected that further research and development efforts will place significant emphasis on verifying the data format integrity. Figure 1 shows a view of the overall performance of the proposed framework, displaying the role of techniques such as edge intelligence and deep learning in our framework. Edge intelligence can be used to process real-time data and provide meaningful insights. Deep learning can be used to analyze the data and provide more accurate predictions. Both techniques are essential for successful implementation of the proposed framework.

Sports can be employed to assess PPG signals, while we acknowledge that supplementary physical testing can yield more precise results. In comparison to objects positioned directly in front of the body, these are much easier to manage. The present technique has successfully achieved commercial accuracy for the evaluation model. Broadly speaking, this study emphasizes many essential aspects:

1. This study presents a novel advanced deep learning architecture designed to forecast fatigue levels in student athletes after workouts. In addition, we offer a novel approach that integrates many data sources using a multi-task learning mechanism. Finally, we verified the technique using empirical data, and the results demonstrate its precise assessment of student fatigue during sports activities. This is accomplished through the utilization of an edge intelligence platform and the application of deep learning algorithms to PPG signals.
2. A computational model capable of quantifying fatigue probability during a sports medical evaluation. The model utilizes Bidirectional Long Short-Term Memory (BILSTM) and an enhanced residual network (ResNet). Additionally, a distinct Xception model architecture incorporating BILSTM is employed to detect sports-induced fatigue in students by analyzing PPG data. The evaluation of this model on the sports physical exam dataset reveals that ResNet-BiLSTM outperforms other models in predicting weariness results. In addition, we assess the Xception-BiLSTM model and observe that it exhibits comparable performance to the ResNet-BiLSTM model.
3. The existing approaches to monitoring students’ physical activity are hindered by insufficient data processing and imprecise computational models. While our initial dataset comprised 12 participants, we employed a data augmentation technique called windowing, increasing our sample size. This substantial augmentation enabled us to train and validate our models more effectively, enhancing their ability to generalize across different conditions and subjects. The augmented dataset allowed for rigorous validation using a fivefold cross-validation approach, demonstrating a classification accuracy of 91.8%, thus underscoring the robustness and effectiveness of our model. In this study, we employed PPG signals to classify participants as weary or non-fatigued. A comparative study is being done to evaluate deep learning models. Identifying the optimal PPG parameters with limited training data is a really difficult task. To address this challenge, we propose an effective approach to enhancing deep learning models by using simulated PPG signals. This method allows parameter optimization by decreasing model computational complexity. The proposed technique can improve fatigue evaluation precision based on PPGs.

The following sections of this manuscript are arranged in the following order. An overview of existing research on PPG readings is provided in the following section. Several deep learning models were used in the analysis, including LSTM, Xception, ResNetCNN with Bidirectional LSTM (BILSTM), and Xception with BILSTM. “Proposed method” section provides a detailed description of the architectures of these models. The study’s “Results” section presents a comprehensive analysis of the outcomes obtained from a number of deep learning models. “Conclusion” section presents the paper’s conclusion.

## Related work

There has been a limited amount of research conducted on the issue of detecting fatigue in pupils using PPG or physiological signs. Nevertheless, extensive scholarly investigation has been conducted, and this particular body of research is frequently cited to address the phenomenon of fatigue experienced after physical exertion.

Objective physiological indicators are useful for assessing fatigue because they yield quantifiable insights into the biological response. Hence, these methodologies serve as a valuable supplement to subjective evaluations and are crucial for comprehending the intricate correlation between physiological changes and fatigue. In order to assess fatigue utilizing objective physiological markers, there are a variety of approaches to quantifying the physiological response to exhaustion. In addition to complementing subjective assessments, these methods provide invaluable insight into the complex relationship between weariness and physiological shifts. In the following, other studies examine some concrete solutions for identifying fatigue from physiological signals. The advantages and disadvantages of each are discussed.

### Heart rate variability

Numerous methodologies employ HRV to measure heart rate variability^5–7^. HRV can be utilized as an indicator of autonomic nervous system (ANS) activity through the analysis of inter-beat intervals. Depletion has an effect on autonomic function, and HRV is a sensitive measure of both tiredness and regulation dynamics. Another approach measures how the skin’s electrical conductivity changes in response to psychological and physiological stress. Skin conductance may indicate how exhaustion affects the body’s physiological stress reaction. Breathing, exercise, drug use (especially caffeine and narcotics), stress, mood, and preexisting illnesses can skew HRV readings^9–14^. HRV results can be affected by movement, exercise intensity, and breathing patterns-particularly deep or fast breathing. Variables such as these introduce variability into HRV measurements.

The main advantage of the study by Al-Libawy et al.^5^ is that it provides accurate feedback to operators about their level of fatigue. Additionally, it offers valuable insights into managing operator fatigue. However, the study has some limitations, such as the need for operators to wear a wearable sensor, which they may be unwilling to do.

The study by Bekhouche et al.^6^ has the advantage of using deep learning to identify drowsiness in drivers, a powerful and accurate technique. However, its disadvantages include being computationally expensive and requiring a large amount of data to train the model.

The study by Karthikeyan et al.^7^ has several advantages, including the ability to measure stress in real-time, detect stress non-invasively, and use ECG and HRV signals to identify stress. However, the study relies on technology that may be inaccurate in certain situations, and its sample size is small.

HRV is frequently noticed in assessments because of its ability to detect alterations in the autonomic nervous system, which can be influenced by exhaustion. It has been shown that fatigue affects HRV by acting on the autonomic nervous system. When trying to gauge someone’s exhaustion, measuring HRV may be helpful.

### EEG signals

EEG signal can shed light on cognitive, focus, and attention processes^26,27^. Cognitive exhaustion can be detected by analyzing observable abnormalities in EEG rhythms, which can serve as a measure of cognitive fatigue. The brainwave signal analysis includes only a few frequencies, including Alpha, Beta, Delta, and Theta.

Li et al.^26^ and Trejo et al.^27^ highlighted the importance of EEG signals in understanding cognitive, focus, and attention processes. Cognitive exhaustion can be detected by analyzing abnormalities in EEG rhythms, which serve as a measure of cognitive fatigue. The analysis focuses on specific frequencies, including Alpha, Beta, Delta, and Theta.

A major advantage of the study by Li et al.^26^ is its comprehensive analysis of how different workloads affect situational awareness. However, the study’s limitation is that it only used EEG and eye tracking as measures of situational awareness, excluding other important factors such as reaction time, accuracy, and decision-making ability. Trejo et al.^27^ provide a thorough examination of the effects of mental fatigue on EEG signals. The study’s disadvantage is that it classifies mental fatigue based solely on EEG signals, without identifying the underlying causes of mental fatigue.

In the study by Yang and Ren^28^, EEG signals were measured during exercise to determine how fatigue affects them. Machine learning techniques were used to extract features from the EEG signals to simulate fatigue-induced changes. This study can contribute to developing more reliable and accurate digital biomarkers of fatigue by enhancing the understanding of the neurological effects of exercise-induced fatigue. However, the study has several disadvantages, including a small sample size and potential confounding factors such as the environment.

### EMG signals

In the study by Liu et al.^29^, EMG measures the electrical activity of muscles during contractions. By providing objective data on muscle fatigue levels, this method can be valuable in assessing the physical demands and fatigue in sports. An EMG patch was used to monitor muscle fatigue during exercise. This patch provides real-time feedback by measuring muscle electrical activity, allowing users to adjust their exercise intensity and duration to minimize the risk of overworking their muscles. This study introduces a novel method for measuring muscle fatigue in real-time, which can be beneficial in sports, rehabilitation, and other medical fields. However, the EMG patch is still in its early stages and may encounter technical errors. Additionally, there are potential health risks associated with wearing the patch for extended periods.

EMG signals are used in the study by Liu et al.^30^ to detect fatigue and abnormal states during football training. By measuring muscle activity in different parts of the body, EMG signals can identify fatigue and abnormalities, potentially improving both safety and performance. However, EMG signals can be inaccurate, and it is challenging to determine which signals specifically indicate fatigue or abnormalities.

Cui et al.^31^ investigated the effects of training on EMG and physiological parameters of athletes using nano biomechanics analysis to measure changes during various training states. The results provide valuable insights into how athletes respond to training and offer a better understanding of the physiological changes that occur during exercise, which can help optimize training regimens. However, the study is limited by the fact that it was conducted in an in vitro setting.

### ECG signals

PPG and ECG data are utilized to estimate HRV (Heart Rate Variability) in the studies conducted by Karthikeyan et al.^7^ and Wittenberg et al.^32^. ECG records the electrical activity of the heart, offering precise information about the intervals between heartbeats. On the other hand, PPG measures circulation and blood volume by detecting changes in light absorption or reflection by the exposed skin, typically at the fingertip or wrist.

In order to evaluate the level of fatigue in children, the study by Jiang et al.^33^ utilizes the measurement of the heart’s electrical activity during running. An electrocardiogram (ECG) is employed to measure various parameters including heart rate, time intervals, and heart rate variability. It is hoped that this study will offer insights into how running fatigue impacts young athletes, provide accurate measurement methods, and suggest strategies to enhance training and alleviate fatigue.

However, the study is subject to several limitations. Firstly, the sample size is small, and the participants are limited to the same age group of students. Additionally, the study does not account for potential external factors such as nutrition, sleep, or stress, which may also influence fatigue levels. Addressing these limitations could enhance the study’s comprehensiveness and applicability to real-world scenarios.

In the study by Wannenburg et al.^8^, ECGs and PPGs are noted to be susceptible to power noise and electromagnetic interference (EMI), which can complicate the evaluation of Heart Rate Variability (HRV). EMI can contaminate the ECG signal, introducing unnecessary information and making it more challenging for HRV researchers to identify critical peaks. This phenomenon adds another layer of complexity to interval measurement, potentially affecting the accuracy of HRV assessment.

In the study by Zhu et al.^34^, a short-time Fourier transform and convolutional neural network are employed to detect exercise fatigue. The research relies on heart rate data collected from subjects during exercise. According to the study, this method accurately detects fatigue in subjects. One advantage of the study is its reliance on deep learning algorithms, which are known to be more accurate than traditional methods in detecting fatigue. However, drawbacks include the requirement for increased computing power and time during model training and validation.

Wang et al.^35^ aims to develop a real-time fatigue detection application utilizing physiological signals like heart rate, respiratory rate, and body temperature. Deep learning algorithms are employed by the application to detect fatigue. This study suggests that coaches and trainers could enhance their decision-making regarding when to rest or adjust training regimens by detecting fatigue in athletes’ bodies in real-time. However, a major disadvantage is the uncertainty surrounding the long-term effects of deep learning on athletes’ bodies.

### PPG signals

As blood flow decreases, metabolic waste chemicals can build up within blood vessels, activating nerve endings. The situation at hand is characterized by tissue hypoxia or lactic acidosis. During this physiological process, metabolic waste products like lactic acid accumulate in the tissues. The process of accumulation might potentially lead to weariness or discomfort through the activation of pain receptors and disruption of nerve transmission^22,23^. The process of PPG assesses changes in blood volume inside blood vessels, providing vital insight into the health of the cardiovascular system as a whole^24,25^.

Bretonneau et al.^36^ introduced a method for remote photoplethysmography (rPPG) that is resistant to motion artefacts, allowing people to be assessed without direct contact. By using a convolutional neural network (CNN)-based rPPG construction module, they obtained robust rPPG signals. The module consisted of a dual feature extractor that focused on signal-based targets, a base span module, and a noise reduction module.

According to Feng et al.^36^, physiological indications and techniques have shown efficacy. Through the use of ECG data, athletes’ physical fatigue was predicted during rope skipping activity. In order to obtain ECG data during training, the researchers designed a cognitive task that involved training. Using sophisticated ECG capturing equipment called Shimmer3, they examined the patients’ ECGs.

An analysis of heart rate variability signals obtained from PPG and ECG sensors is presented in the study by Królak et al.^37^. PPG sensors can measure heart rate variability accurately and reliably in this study. The non-invasive nature of this study makes it suitable for long-term studies since it does not require physical contact with the subject. Furthermore, PPG sensors are cheaper than ECG sensors and can be embedded in wearable devices. This study has the disadvantage that PPG sensors are less reliable than ECG sensors, and they may be affected by environmental noise.

Guo et al.^38^ developed a fitness evaluation model for teenagers that integrated a one-dimensional Convolutional Neural Network (1D-CNN) and Long Short-Term Memory (LSTM). PPGs were collected from 1024 adolescents using the recommended three-stage running paradigm. After removing noise from the unprocessed data, the wavelet transform and median filter were applied to extract HR and SpO_2_. Using the Pearson correlation coefficient method, they constructed the feature set using the nine physical qualities obtained previously. Finally, a 1D-CNN with LSTM model was developed to classify teenagers’ physical fitness levels into four categories: outstanding, good, medium, and awful. Men’s and female’s physical aptitudes were predicted with 98.27% and 99.26% precision, respectively.

What can be concluded from previous research is that identification of fatigue for students due to exercise has rarely occurred. Few studies use PPG signals. This could be due to the difficulty of accurately measuring fatigue with PPG signals. Further research is needed to explore the potential of PPG signals to accurately measure fatigue in students. In this case, deep learning can solve a significant part of the mentioned challenge. Deep learning models can be trained to recognize patterns in PPG signals that can be used to accurately measure fatigue. This technology can be used to create more effective and personalized solutions to the challenge of fatigue in students.

## Proposed method

**Figure 2:**
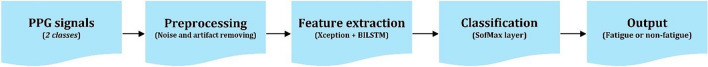
Structure of the suggested assessment system.

As part of our study, we analyzed the fatigue’s physiological data to determine their current level of physical fatigue. In Fig. 2, we present the results of our efforts to develop a method to assess the physical fitness of pupils during sporting activities. PPGs were collected from many students using an experimental paradigm designed specifically for this purpose. The original PPGs were also preprocessed using our techniques, ensuring that the signals were sufficiently purified for further analysis. In the following step, we computed heart rate (HR) and oxygen saturation (SpO2) to derive nine discernible attributes from the processed data. The final decision was based on evaluation criteria. To categorize the children’s levels of physical activity, a refined model assessment framework was developed.

### Noise reduction in PPG signal

Sometimes PPG signals are misinterpreted for other types of sounds. However, the deep structure can serve as a model for feature extraction. Enhancing the process of noise reduction has the ability to boost the effectiveness of the characteristics that are obtained. The frequency domain filtering approach performs inconsistently and may not be able to entirely eliminate baseline wander when applied to non-stationary data. Since Wavelet Transform (WT) denoising approaches have been demonstrated to be more successful than filtering techniques while needing less computer resources, they have been frequently used in physiological signal denoising. The ideal choice of WT parameters—the mother wavelet function, the thresholding function, the amount of decomposition, and the threshold for noise—is necessary for wavelet signal denoising to be effective. Wavelet shrinkage denoising typically involves three distinct processes:

#### Decomposition

The unprocessed, noisy PPG signal is subjected to the Discrete Wavelet Transform (DWT) at a specified decomposition level, denoted as *k*. At each level, the signal is divided into detail coefficients (cD) and approximation coefficients (cA):

1 $${\text{PPG}}_{\text{noisy}}(t)=\sum \limits_{i=1}^{k}\sum \limits_{n\in Z}c{D}_{i}(n){\Psi }_{i,n}(t)+\sum \limits_{n\in Z}c{A}_{k}\left(n\right),$$

2 $$c{D}_{i}\left(m\right)=\left({\text{PPG}}_{\text{noisy}},{\Psi }_{i,m}\right),$$

which is the result,

3 $$c{D}_{i}(m)=\frac{1}{\sqrt{2}}\sum \limits_{n}g\left(2m-n\right)c{D}_{i-1}\left(m\right),$$

moreover,

4 $$c{A}_{k}\left(m\right)=\left({\text{PPG}}_{\text{noisy}},{\varphi }_{k,m}\right),$$

which is the result,

5 $$c{A}_{k}(m)=\frac{1}{\sqrt{2}}\sum \limits_{n}h\left(2m-n\right)c{A}_{L-1}\left(m\right).$$

At level *i*, individual coefficients are denoted by *cD*_*i*_(*n*), i = 1,2,…,k, while at level *k*, approximation coefficients are denoted by *cA*_*k*_(*n*). In the wavelet filter bank, the low pass filter is represented by the symbol h(n), whereas the high pass filter is represented by the symbol g(n).

#### Thresholding

With advanced threshold selection techniques like Minimax, Stein’s Unbiased Risk Estimation (SURE), or the universal approach, we can accurately determine the noise variance of the detailed coefficients (*σ*_*i*_, i = 1,2,…,*k*) at each level. Based on this information, the threshold value for each level is calculated (*γ*_*i*_, i = 1,2,…,*k*). Through thresholding procedures, the various coefficients are then constrained to the predetermined threshold values. The hard thresholding method involves the following steps:

6 $$c{D}_{{i}{\prime},n}=\left\{\begin{array}{cc}0& \left|{D}_{i,n}\right|\le {\gamma }_{j}\\ c{D}_{i,n}& \left|{D}_{i,n}\right|\ge {\gamma }_{j}\end{array}\right.,$$

moreover, the soft thresholding is defined as:

7 $$c{D}_{{i}{\prime},n}=\left\{\begin{array}{cc}0& \left|{D}_{i,n}\right|\le {\gamma }_{j}\\ \text{sgn}(c{D}_{i,n})(\left|c{D}_{i,n}\right|-{\gamma }_{i})& \left|{D}_{i,n}\right|\ge {\gamma }_{j}\end{array}\right.,$$

where, the level *j* contains the noisy detailed coefficients, which are represented by *cD*_*i,n*_. On the other hand, the denoised detailed coefficients at index n are marked by ${\text{cD}}{\text{CD}}_{\acute{i,n}}$.

#### Reconstruction

Inverse Discrete Wavelet Transform (IDWT) *k*th approximation coefficients and *k* detail coefficients are used to reconstruct the denoised PPG signal:

8 $${\text{PPF}}_{\text{denoised}}\left(t\right)=\sum \limits_{i=1}^{k}\sum \limits_{n\in Z}c{D}_{{i}{\prime}}\left(n\right){\varphi }_{i,n}\left(t\right)+{\sum \limits_{n\in Z}c{A}_{k}\left(n\right)\varphi }_{k,n}\left(t\right).$$

### ResNet and Xception

The model incorporates ResNet networks^39^, where one network analyzes input spectrogram images obtained from PPG signals that are associated with the student’s athletic activity, while the second network analyzes reference images that are also connected to the same activity. Incorporating two more layers has the potential to improve the surface characteristics. A convolution is used to convert many spectrogram images into a cohesive feature mapping. An alternative approach of describing the conclusion is as follows:

9 $${x}_{j}^{{\ell}}=f\left(\sum \limits_{i\in {M}_{j}}{x}_{j}^{{\ell}-1}\times {k}_{ij}^{{\ell}}+{b}_{j}^{{\ell}}\right).$$

The convolution kernel is represented as *k*_*ij*_, whereas the *ℓ* layer is marked as *ℓ*. *M*_*j*_ denotes the set of input mappings, whereas *b*_*j*_ indicates the bias. Another example illustrating the application of the sigmoidal function is as follows:

10 $${v}_{ij}^{xy}=\text{sig}\left({b}_{ij}+\sum \limits_{p=0}^{{P}_{i}-1}\sum \limits_{q=0}^{{Q}_{i}-1}{w}_{ij}^{xy}{v}_{(i-1)}^{(x+p)(y+q)}\right).$$

In this context, *v*_*ij*_^*xy*^ indicates a value where *v*_*i*_ represents the *j*_*th*_ characteristic, and *x* and *y* identify the coordinates of the *i*_*th*_ level. The weight of the kernel is denoted as *w*_*ij*_^*pq*^, with *Q*_*j*_ representing its width and *P*_*i*_ representing its height. When doing bias mapping, it is necessary to employ the functions *b*_*ij*_ and sig(.). Consequently, a layer of stochastic integration was employed. As the variability decreases and the maximum value of a significant characteristic is identified in spectrogram pictures, the practical application of the stochastic integration layer becomes apparent. To address the problem of over-fitting, one might employ the previously indicated layers. When adapting the deep transfer learning architecture of CNN models, the biases and weights are determined based on the following factors:

11 $$\Delta {W}_{l}\left(t+1\right)=-\frac{x\lambda }{r}{W}_{l}-\frac{x}{n}\frac{\partial C}{\partial {W}_{l}}+m\Delta {W}_{l}\left(t\right),$$

12 $$\Delta {B}_{l}\left(t+1\right)=-\frac{x}{n}\frac{\partial C}{\partial {B}_{l}}+m\Delta {B}_{l}\left(t\right).$$

*C*, *t*, *m*, *n*, *x*, *l*, *B*, and *W* represent the cost function, update step, momentum, learning rate, regularization parameter, thickness, bias, and weight, respectively, in that specific order.

The Xception is a type of deep neural network that uses depthwise separable convolutions to make the learning process more efficient and better at recognizing patterns. Depthwise separable convolutions reduce the computational cost of training the network, allowing it to learn more efficiently and accurately. Xception networks are also capable of recognizing complex patterns in data, making them ideal for tasks such as image classification and object detection. Additionally, these networks are easier to train and integrate into existing deep learning models.

Xception networks can be used to detect patterns in PPG signals that may indicate fatigue. For example, they can detect changes in heart rate, respiration rate, and other vital signs that may be indicative of fatigue. Additionally, Xception networks can also be used to detect changes in posture, muscle activity, and other physical indicators that may be indicative of fatigue. Xception networks are able to learn complex relationships between different features, allowing them to detect subtle changes in the PPG signals during sport. This makes them suitable for fatigue detection, as small changes in PPG signals can be indicative of different levels of fatigue. Additionally, Xception networks are relatively easy to train and integrate into existing deep learning models, making them a good option for the task of fatigue detection in PPG signals during sport.

### Xception with BILSTM

**Figure 3:**
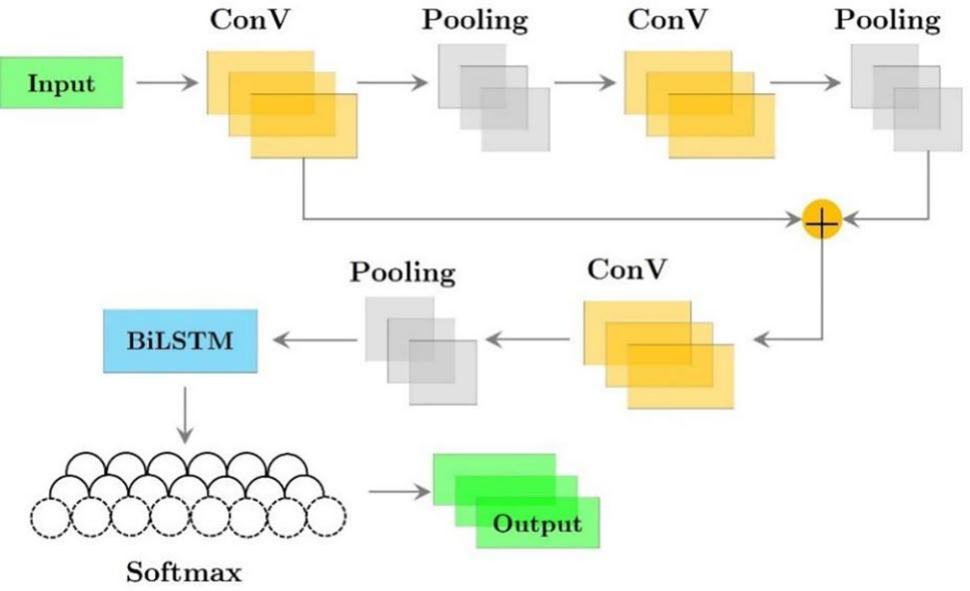
Integrating ResNetCNN and BILSTM into a deep learning framework.

**Figure 4:**
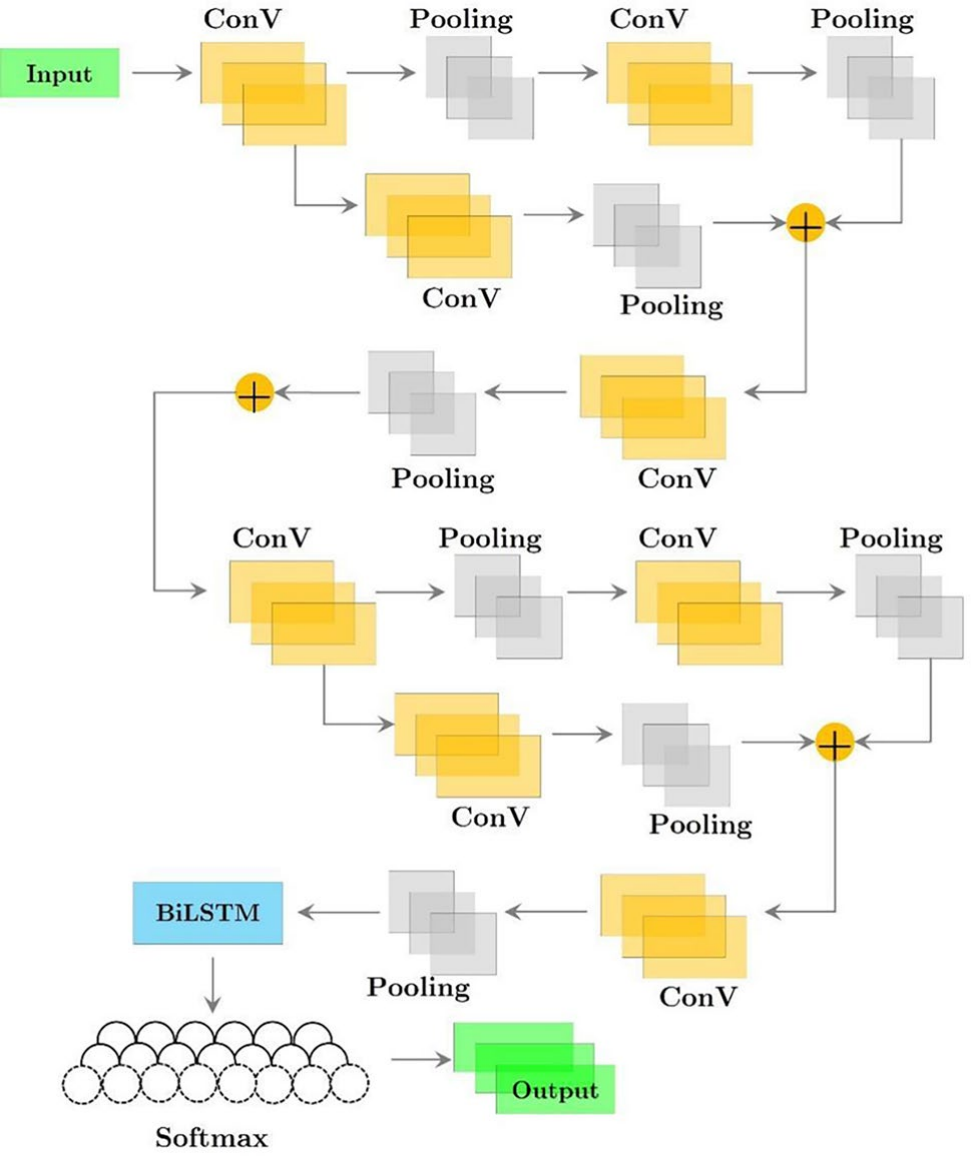
A deep learning architecture combining Xception and BILSTM has been suggested.

A component of the experimental procedure included time series data derived from PPG. The correlation level exhibited by permutation sequences strongly influences time series data models. To improve classification accuracy, it is imperative to simultaneously extract the temporal information embedded in time series data and the wave attributes inherent in the spatial domain of the measured signal. ResNetCNN with BILSTM (Fig. 3) and Xception with BILSTM (Fig. 4) were compared in this study. Both models were evaluated using the same dataset to categorize fatigue in students due to sports.

According to the database records, each input contained several data points. Based on a variety of indications, each of these extensive data sets is capable of generating two to three PPG waves. Recent studies have demonstrated that ResNetCNN models in conjunction with BILSTM models are highly effective in analyzing PPG data. Under the constraints of limited PPG training data, this research investigates ideal parameters, such as the kernel, kernel size, and layers. The parameters that have been determined for our proposed model, which combines the Xception architecture with BILSTM, are also described in detail. When applied to the PPG database, this configuration should maintain a constant level of accuracy.

### ResNetCNN with BILSTM

ResNetCNN with BILSTM was used to obtain feature vectors from the input data, which had a 36-layer ResNetCNN architecture. As a result of the extraction technique, each data point yielded a characteristic vector with dimensions of 15 × 132. Following feature extraction, the characteristic vectors were used as inputs to a Bidirectional Long-Short-Term Memory (BILSTM) model. As a result, the computer acquired knowledge of the temporal relationship between the extracted characteristics. The Xception with BILSTM model extracted features from the input data using the 37-layer Xception architecture. Each data point was extracted as a 64 × 132.

The characteristic values were then input into a Bidirectional Long-Short-Term Memory (BILSTM) model to facilitate the computer’s understanding of the temporal relationship between these elements. PPG data were categorized in this study to enhance understanding of hypertensive illness diagnostic outcomes. Therefore, the loss function for neural networks was determined to be cross entropy loss. In Algorithm 1, pseudo-code of ResNetCNN with BILSTM method is displayed.

Algorithm 1—It is presented here the pseudocode for ResNetCNN-BILSTM.

**Algorithm 1:**
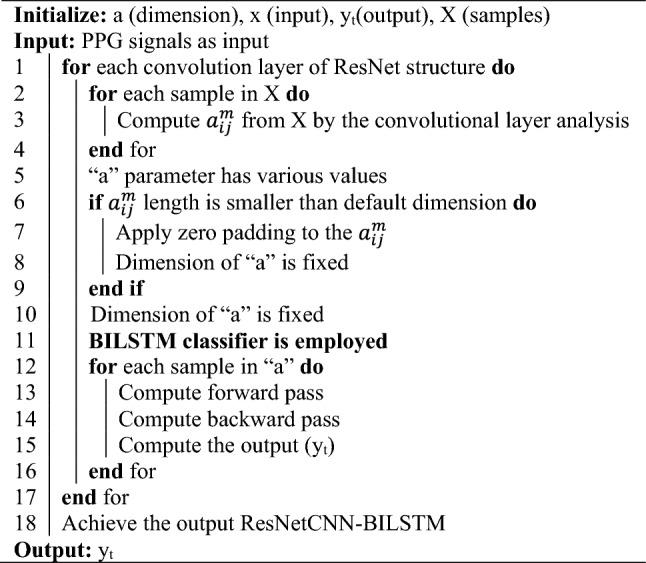
ResNetCNN-BILSTM procedure.

### Classification

Softmax assigns probabilities to each element in vector z based on the exponential of the input numbers, resulting in a direct proportionality. Before using the softmax function, it is possible for certain components of the vector to have negative values or have multiple elements, resulting in a sum of zero. A Softmax function guarantees that every variable, when transformed into a probability, falls within the range of 0 to 1, and that the sum of these probabilities is exactly 1. The likelihood of success increases in direct proportion to the accomplishment’s scale. A typical Softmax function $\sigma \text{:}{\mathbb{R}}\to {(0,1)}^{\text{K}}$ can be defined as follows when K ≥ 1 is provided:

13 $$\sigma (z{)}_{i}=\frac{{e}^{{z}_{i}}}{\sum \limits_{j=1}^{K}{e}^{{z}_{j}}} \text{ for }i=1,...,K,$$

where, *z* is described as (6):

14 $${z=({z}_{1},\dots ,{z}_{K})\in {\mathbb{R}}}^{K}.$$

We apply the exponential function to each member *z*_*i*_ of an input vector *z* to elucidate. Next, to get the standard vector, we divide the result by the sum of all exponential functions. The output vector (*z*) is normalized when all of its components are changed such that their total is 1. In addition, the mathematical constant *e* can be substituted with any base having a value larger than zero. The result is dependent on b, which must be between zero and one. Lower values of *b* cause probabilities to be more concentrated on the regions with the smallest input values, whereas larger input components are correlated with greater output probabilities. It is more probable that a larger output will be produced when the variable b grows when the size of the input component increases. A probability distribution that is skewed towards the maximum input values is also produced by *a* bigger *b*. We may use the statement above to find *σ(z)*, where *β* is a real integer and *b* is defined as either α_1_ or α_2_^40^.

15 $$\sigma (z{)}_{i}=\frac{{e}^{\beta {z}_{i}}}{\sum \limits_{j=1}^{K}{e}^{\beta {z}_{j}}} \text{ for }i=1,...,K.$$

In certain domains, the base or scale remains constant, but in others, the *β* can fluctuate.

## Results

### PPG signals

The analyzed data was done according to the protocol presented in the research^41^ and we used a large number of students in the analysis. This study collected data to evaluate the effectiveness of the suggested method in detecting weariness caused by learning in a real-life setting, without interfering with students’ regular activities. The research procedure was officially endorsed by the University Ethics Committee. The experiment was conducted in two separate settings: (1) a university classroom with fluorescent lights hanging from the ceiling, without any ability to adjust their brightness, and (2) during periods of intense studying by students, which was anticipated to lead to mental fatigue. A total of 12 physically fit students participated in the data gathering for this study. Every student had to undertake two separate examinations: one while experiencing great exhaustion and another under normal circumstances. Each examination was assigned a time frame of 10 min.

In order to evaluate the students’ current level of learning prior to each test, we utilized two assessment tools. The first tool was the 2-min Psychomotor Vigilance Test (PVT), which measures instances of decreased attention caused by fatigue^42^. The second tool was the Karolinska sleepiness scale (KSS) form, a commonly employed method for detecting sleepiness^43–47^. The corresponding results can be displayed in Table 1. The mean latency, measured in milliseconds (ms), is presented in the third and fifth columns of Table 2. The mean ± std denotes the average and standard deviation (std) for all participants in both the fatigue and non-fatigue tests.

**Table 1:** Auto-evaluation form for assessing fatigue using the Karolinska Sleepiness Scale (KSS).

Scale	Class	KSS factor
6	Fatigue	Very sleepy
5	Fatigue	Sleepy in an effort to awake
4	Fatigue	Sleepy
3	Non-fatigue	Alert
2	Non-fatigue	Very alert
1	Non-fatigue	Extremely alert

**Table 2:** This table demonstrate of PVT and KSS results^42^.

No. of participants	Fatigue		Non-fatigue	
	KSS	PVT (ms)	KSS	PVT (ms)
S_01_	7	553	2	365
S_02_	8	388	3	285
S_03_	7	404	3	324
S_04_	7	424	3	363
S_05_	7	447	3	365
S_06_	7	399	3	350
S_07_	8	386	3	346
S_08_	7	511	3	352
S_09_	9	532	3	393
S_10_	8	350	2	398
S_11_	7	646	3	362
S_12_	7	593	3	372
Mean ± STD	7.4 ± 0.6	469.4 ± 87.2	2.8 ± 0.3	356 ± 28.5

### System setting

To evaluate the model’s ability to handle several states, the recommended classifier was used. We divided the data into two segments: training and testing. We used k-fold cross-validation (CV) to evaluate the algorithm’s performance and create validation data, specifically a fivefold CV separated by subject (student).

Initially, our data consisted of recordings from 12 participants. However, we performed windowing on this data, where the recordings were segmented into smaller windows. This windowing process effectively augmented our dataset, resulting in up to 450 windows for some participants. To ensure the validity of our cross-validation process, we applied a fivefold cross-validation strategy on these windows. Here’s how it worked:

1. Each participant’s signal was divided into multiple small windows. For instance, if a participant’s signal was divided into 40 windows, these windows were used in the CV process.
2. The classification algorithm was then applied to these windows. If the majority of these windows for a specific participant were classified as indicating fatigue, the entire signal for that participant was labeled as fatigue.

This approach allows us to increase the number of samples significantly, providing a more robust dataset for training and evaluation. It also ensures that the cross-validation is performed in a manner that respects the separation of subjects while leveraging the augmented data for better model performance.

PPG signals previously unobserved were compared to the trained model with optimized parameters. Segmented signals were used for decision-making by labeling each window and assigning the predominant label to each signal within the windowed PPG sample. The decision-making process can use the most commonly occurring label assigned to each signal if each window in a sample of windowed PPG data is labelled. Computer components equipped with 64-bit operating systems were used in the development of our solution. CPUs with Core i7 graphics cards and 8 Gigabytes of Random Access Memory (RAM). Initially, this design acquired knowledge at 0.001. For effective learning, 500 to 2000 epochs were recommended. Stochastic Gradient Descent (SGD) significantly improved the performance of the proposed architecture. In order to deploy a hybrid model in production, it must be trained on a single CPU for up to 6 h. Structures recommended by the experts are incorporated into our designs. Four to 6 h are required for each iteration of training and optimizing CNN models. Clinical system design, testing, convergence, training protocols, error estimations, and fine-tuning are critical components of successful validation and training processes. By minimizing training and validation errors, we achieved optimal convergence for each convolutional CNN architecture. In the event that accuracy tests consistently fail or error rates do not decrease, training is immediately stopped.

### Model setting

With a small quantity of training data, the study aims to determine the most effective model and parameters. Three types of parameters can be modified in various deep learning models: layers, kernels, and kernel size. We anticipate that performance will fluctuate based on both temporal and spatial factors due to the decrease in training continuous PPG signals. We augment the BILSTM model with either ResNetCNN or Xception in order to improve precision. Additionally, BILSTM, ResNetCNN, and Xception networks were used as deep learning models in this study. Data characteristics were obtained using ResNetCNN and Xception networks, each of which was trained with a separate setup.

A one-layer BILSTM was used to investigate the temporal transmission characteristics of the PPG wave. Table 3 shows the simulated parameters. For one-dimensional data, we evaluate both spread and deep models. Among many models, we prioritize identifying the most effective parameter combinations. Both ResNetCNN and Xception parameter sets are included in the tables. With limited training data, the main goal is to determine the optimal number of layers and to select a good kernel. Various parameters were used in this study to compare the classification efficacy of ResNetCNN.

**Table 3:** Modifications in Xception variables for the development of a decision-making model.

Xception	No. layers	Kernels	Kernels size
Setting 1	31	30	34
Setting 2	34	31	35
Setting 3	37	32	36
Setting 4	40	33	37
Setting 5	43	34	38

At layer 31, the study specifically analyzed the performance of ResNetCNN with different layer configurations (21, 26, 31, 36, and 41) and kernel sizes (13, 14, 15, 16, and 17). Additionally, the study evaluated the performance when the kernel size was 15 and the layers were 31, with kernel sizes ranging from 37 to 41. This comparison evaluated the performance of neural networks when operating with less data. Aim of this study was to determine the most effective network parameter for extracting characteristics from data. A number of parameter configurations were used in this study to assess Xception’s categorization skills. Layers = 31, 34, 37, 40, and 43; kernel = 30, 31, 32, 33, and 34 at layers = 37; and kernel = 32 and layers = 34, 35, 36, 37, 38.

### Assessments and comparison

An advanced deep learning model utilizing a multi-interest network is proposed. Four performance metrics are examined in this study: F1-measure, Recall, Root Mean Squared Error (RMSE), and Mean Absolute Error (MAE). Prediction precision can be evaluated using recall and the F1-measure. PPG signals are analyzed using a portion of the available data. A system’s prospective effectiveness can also be assessed using the F1-measure, a statistical technique. A calculation of the average of precision and recall scores is used to make the determination. Under a threshold value K, accuracy (Pr_K_) is the proportion of suggestions that are correct compared to the total number of suggestions. F1(K) is sometimes referred to as F1-measure, and it is defined as follows:

16 $${F}_{1(K)}=2\times \frac{{\text{Pr}}_{K}\times {\text{Re}}_{K}}{{\text{Pr}}_{K}+{\text{Re}}_{K}}.$$

By using two distinct measures, statistical analysis evaluates the accuracy of prediction systems. It is possible to determine the absolute inaccuracy of $|{\widehat{\text{r}}}_{{\text{u}}_{\text{i}}}-{\text{r}}_{{\text{u}}_{\text{i}}}|$ by comparing the projected rating ${\widehat{r}}_{{u}_{i}}$ with the actual rating r_ui_ from the Test collection. Here, the quantity of tests is represented by |Test|. All combined test results are represented by the mean absolute error (MAE). To obtain the standard deviation of all test instances, use the root-mean-square error (RMSE). By reducing RMAE or MSE, idea development shows enhanced performance.

17 $${\text{MSE}} = \mathop \sum \limits_{{r_{ui} \in {\text{Test}}}} \frac{{\left( {\hat{r}_{{u_{i} }} - r_{{u_{i} }} } \right)^{2} }}{{\left| {{\text{Test}}} \right|}}.$$

In addition,

18 $${\text{MAE}} = \mathop \sum \limits_{{r_{ui} \in {\text{Test}}}} \frac{{\left| {\hat{r}_{{u_{i} }} - r_{{u_{i} }} } \right|}}{{\left| {{\text{Test}}} \right|}}.$$

Moreover, we define that MSE = (RMSE)^2^. A decision-making model can be developed using similar methods as shown in Table 4. As well as focus groups and criteria of data analysis, Xcepetion, ResNet, and a combination of these two architecture methods were used.

**Table 4:** Results of similar methods are shown for the development of a decision-making model.

Model	Accuracy (%)	Recall (%)	Precision (%)	MAE	RMSE
Xception	85.6	81	84.5	2.431	3.674
ResNetCNN	87.2	84	87.6	2.326	3.449
LSTM	86.4	83	85.8	2.414	3.563
Xception with BILSTM	90.3	89	89.4	2.069	3.223
ResNetCNN with BILSTM	91.5	89.5	89.7	2.012	3.019

Learning fatigue detection is validated by performing fivefold (subject-dependent) cross-validations on the dataset obtained by the person. Training and assessment are conducted on a sample of 11 individuals and a separate individual, resulting in a total of 12 participants. 440 samples are used for training, and 40 samples are used for testing. The process is iterated 11 times using new individuals. There are four performance metrics in Table 5: area under the curve (AUC), F1-score, accuracy, and precision. Based on the four measurements in the last row of Table 5, the suggested technique accurately detects learning fatigue. A proposed approach may also be able to determine if an individual is fatigued or not based on data from other individuals. In order to do so, we consider all four metrics above a threshold of 0.825 in the last row of Table 5. The threshold value of 0.825 was determined through a combination of empirical analysis and optimization techniques.

**Table 5:** This table displays the outcome of a fivefold cross-validation.

Model	Signals	AUC	F1-measure	Precision	Accuracy
^48^	PPG	0.603	0.601	0.607	0.605
^49^	PPG	0.672	0.679	0.692	0.678
^41^	Heartbeats (HR)	0.875	0.872	0.872	0.872
	Breathing (BR)	0.635	0.635	0.635	0.636
	HR and BR	0.902	0.900	0.904	0.901
Xception with BILSTM	Heartbeats (HR)	0.896	0.887	0.890	0.892
	Breathing (BR)	0.756	0.743	0.748	0.746
	HR and BR	0.913	0.908	0.909	0.908
ResNetCNN with BILSTM	Heartbeats (HR)	0.909	0.906	0.907	0.905
	Breathing (BR)	0.854	0.850	0.853	0.851
	HR and BR	0.918	0.912	0.916	0.914

In addition, a comparison of several deep models can be seen in Table 5. First of all, the suggested feature set outperforms the two most advanced feature sets^47,48^. Moreover, the proposed strategy performs better than the other approaches that use single data. As shown in Table 5, the suggested method achieves an accuracy of 0.918, which is much higher than the accuracy of the other individual data methods.

### Discussion

PPG data was analyzed using several deep learning models, including LSTM, ResNetCNN, Xception, ResNetCNN with BILSTM, and Xception with BILSTM. Additionally, it analyzed how time series and geographical characteristics affect network structures. In this study, various parameters were used to compare the network performance of ResNetCNN and Xception with BILSTM. After 500 iterations, the precision of features derived from ResNetCNN with BILSTM or Xception with BILSTM exceeded that of ResNetCNN, Xception, or LSTM individually. The results of several models are shown in Table 5. Fatigue detection was reliably identified in the assessed datasets regardless of whether ResNetCNN or Xception were used individually. The LSTM network design, however, did not provide adequate resolution performance. Xception, ResNetCNN, and BILSTM are presented in Table 5. While ResNetCNN achieves 89% accuracy rate, its recall and precision are considered insufficient.

**Figure 5:**
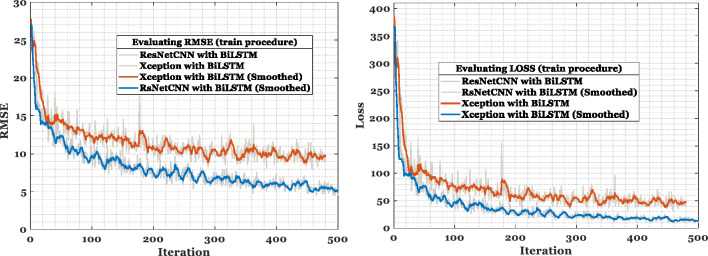
Based on the model’s training phase, fatigue during sport activity is identified from PPG signals using RMSE and Loss criteria.

After 500 training epochs, both the validation set and the model’s accuracy stabilized. Figure 5 shows the same simulation parameters. For the training datasets, Fig. 5 shows the loss, or mean squared error. Due to the substantial amount of interference detected in the study’s unfiltered data, the open dataset yielded disappointing results. In spite of this, the study proved to be successful in validating the Xception with BILSTM model in terms of its overall accuracy.

**Figure 6:**
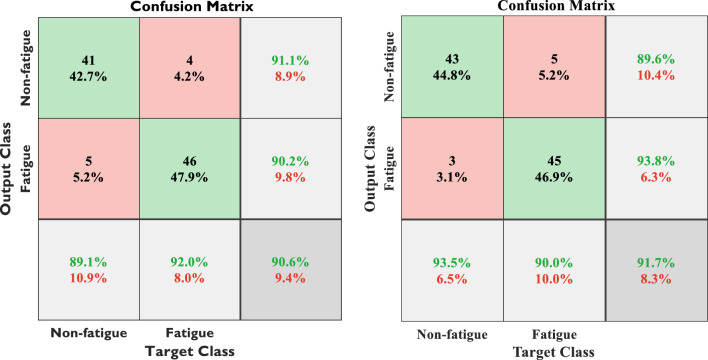
During sports activities, fatigue identification matrices of students can be confused in two ways. These images represent Xception with BILSTM and ResNetCNN with BILSTM structures, respectively, in left-to-right order.

As shown in Fig. 6, multiple techniques provide precise categorization of student exhaustion during physical activity, including both fatigue and non-fatigue situations as confusion matrix. A total of 37 Xception layers are used in the BILSTM model, with a kernel size of 32, a kernel size of 36, and a kernel size of 36. When data is imbalanced, deep learning systems may have difficulty classifying it.

PPGs were used as an indicator of students’ fatigue levels following physical activity in this study. Regardless of whether the activity was intense or low-intensity, the PPGs obtained from the students’ wrists were reliable indicators of their physical activity levels in sports.

Intermediate health levels may be more challenging to assess. Using advanced artificial intelligence methods, students can assess their physical fatigue based on the results of the experiment. In order to compile physiological characteristics for pupils, mathematical computations, noise reduction, and model assessments were used. We used ResNetCNN with BILSTM and Xception with BILSTM, taking into account the one-dimensional nature and temporal correlation of physiological signals, computational capacity, and deep learning’s ability to reduce dimensions. 91.8% of the forecasts were successful.

Data were collected using a standard running test, which may not capture all physiological recordings of students’ physical activity. According to official government documents, this limitation should be considered when predicting their health condition. Despite our acknowledgement that further physical tests can yield more reliable results, long-distance running can be used to assess cardiopulmonary function and is easier to supervise than activities that require sitting in front flexion. For the evaluation model, the current technique has successfully achieved a commercial level of accuracy. In the future, the quality of the model will remain uncertain if signals from other sports are incorporated. Overall, this paper focused primarily on the following topics:

1. As a result of our investigation of the relationship between students’ physiological data and their levels of physical activity during moments of exhaustion, we believe that some key factors can be useful for predicting when students will feel tired.
2. We developed a model for predicting fatigue outcomes in a running physical test. The model uses an enhanced ResNetCNN with BILSTM. By using the proposed model in biosensing recordings, additional predictive tasks might be possible.
3. Results of the experiment demonstrate the feasibility of predicting students’ fatigue levels using biosensing data.

Moreover, by leveraging edge computing, the algorithm can analyze physiological signals such as heart rate, respiratory rate, and body temperature directly on the device where the data is collected from smartwatches, which serve as the primary sensors. This significantly reduces latency and ensures immediate feedback. While the smartwatches function as local processors analyzing fatigue conditions, edge computing acts as an intermediate layer in cloud environments to handle the substantial data received from the smartwatches. This processing not only enhances the system’s responsiveness but also reduces the need for constant connectivity to cloud servers, preserving bandwidth and improving data privacy. In educational settings, where timely detection and intervention are critical, edge computing ensures continuous and efficient monitoring of fatigue, enabling educators and students to make informed decisions promptly. This approach is especially beneficial in environments with limited or unreliable internet access, ensuring the reliability and robustness of the fatigue detection system.

Our paper presents a novel edge computing algorithm designed specifically for the real-time detection of fatigue in students. This algorithm leverages hybrid deep learning models that combine CNN, Xception architecture, and BILSTM architectures to process and analyze physiological signals such as heart rate, respiratory rate, and body temperature.

By deploying the algorithm on edge devices, we ensure low-latency processing and immediate feedback, which is crucial in a real-time educational environment. This approach also reduces dependency on cloud resources, enhancing data privacy and reducing network bandwidth usage.

The hybrid deep learning models were rigorously validated using a comprehensive dataset, demonstrating high accuracy and robustness in detecting fatigue states. The use of CNN, Xception architecture, and BILSTM architectures allows the model to capture both spatial and temporal features from the physiological data, improving overall detection performance.

Our study’s findings have immediate practical implications for improving health monitoring and training regimens in educational settings. The ability to detect fatigue accurately and in real-time can significantly enhance the safety and performance of young athletes. Future work will focus on expanding the participant base and collaborating with other institutions to gather more extensive datasets, further solidifying the generalizability and applicability of our findings.

## Conclusion

Excessive fatigue can significantly reduce learning effectiveness. As far as we know, there is no system capable of acquiring person-independent characteristics for detecting fatigue, i.e., capable of transferring the detection ability, or accurately identifying learning fatigue without specific interventions. PPGs were used in this study to detect signs of weariness in students during sports activities. For fatigue categorization, the PPG approach has proven to be one of the most significant breakthroughs. Using this method, biological signals can be continuously monitored without physical contact by integrating non-contact detection with physiological signal-based detection. In addition, it eliminates the possibility of purposefully disguised emotional artifacts. In addition, we demonstrated that combining deep convolutional neural network (DCNN) techniques can significantly improve fatigue detection accuracy. For deep learning-based classification, it is proposed to use ResNetCNN with BILSTM and Xception with BILSTM architectures. This results in a very precise and adaptable system for detecting student fatigue during sports. Using this technique, it is possible to detect learning fatigue without requiring any particular apparatus. It is important to note, however, that this study does have some limitations. To identify the causes of exhaustion and develop effective treatments, it is necessary to distinguish between drowsiness and fatigue. By using instruments such as the Swedish Occupational Exhaustion Inventory and Fatigue Severity Scale, this can be accomplished. Physiological and psychological weariness should be distinguished when analyzing instructional situations involving sports participation. Furthermore, employing a deep learning approach with a sufficiently large sample size might enhance fatigue detection accuracy. Furthermore, this study did not consider the impact of encounters with students in intricate learning environments, such as those with uneven lighting, motion distortions, and multiple students. These situations should, however, be thoroughly investigated in future research.

## Data Availability

All datasets used in this study are freely available through the open repositories on the web. All data generated or analyzed during this study are included in this published article and its Supplementary Information Files from Ref.^41^, 10.1007/s10489-023-04926-5.
